# Correlation between the severity of coronary artery lesions and levels of estrogen, hs-CRP and MMP-9

**DOI:** 10.3892/etm.2014.1565

**Published:** 2014-02-20

**Authors:** CHANGLEI GUO, SHAOLI ZHANG, JUNBIAO ZHANG, HUI LIU, PEICHENG LI, HENGDAO LIU, YAKUN WANG

**Affiliations:** Department of Cardiovascular Internal Medicine, The First Affiliated Hospital of Xinxiang Medical University, Weihui, Henan 453100, P.R. China

**Keywords:** estrogen, high-sensitivity C-reactive protein, matrix metalloproteinase-9, acute coronary syndrome

## Abstract

The aim of this study was to investigate the correlation between the severity of coronary artery lesions in patients with acute coronary syndromes (ACS) and levels of estrogen, high-sensitivity C-reactive protein (hs-CRP) and matrix metalloproteinase-9 (MMP-9). A total of 65 patients with ACS, 33 patients with stable angina pectoris (SAP) and 36 healthy controls were randomly enrolled. Patients with ACS were subdivided into two groups: Acute myocardial infarction (AMI; n=30) and unstable angina pectoris (UAP; n=35). Serum levels of estrogen, hs-CRP and MMP-9 were detected in the four groups of subjects. Serum estrogen levels in patients with AMI, UAP and SAP were significantly lower than those in the control group (P<0.05). Estrogen levels were also significantly different among the AMI, UAP and SAP groups (P<0.05), with a progressive increase across the three respective groups. Compared with healthy subjects, patients with AMI had the highest levels of hs-CRP and MMP-9, followed in descending order by those with UAP and SAP (P<0.05). Levels of hs-CRP and MMP-9 were also significantly different among the AMI, UAP and SAP groups (P<0.05). Serum estrogen levels were negatively correlated with hs-CRP and MMP-9 levels (r=−0.6634 and −0.6878, respectively; both P<0.05). hs-CRP and MMP-9 levels correlated positively (r=0.7208, P<0.05). The number of stenosed coronary vessels was negatively correlated with estrogen levels (r=−0.6467, P<0.05), and positively correlated with hs-CRP and MMP-9 levels (r=0.6519 and 0.6835, respectively; both P<0.05). In conclusion, serum estrogen, hs-CRP and MMP-9 levels were significantly correlated with the severity of coronary artery lesions. There was also a significant correlation between serum estrogen, hs-CRP and MMP-9 levels. These data indicate that serum estrogen, hs-CRP and MMP-9 have the potential to be used as biomarkers for evaluating the severity of coronary artery lesions and the stability of coronary artery plaques.

## Introduction

Acute coronary syndrome (ACS), one of the most common severe cardiovascular diseases, is a type of severe coronary heart disease that is associated with high rates of mortality and disability (1). ACS is classified as either acute myocardial infarction (AMI) or unstable angina pectoris (UAP), and the classification is based on vulnerable plaque rupture accompanied by complete or incomplete artery occlusion (2). Evaluation of the characteristics of coronary atherosclerotic plaques and the extent of coronary artery lesions can enable early diagnosis of ACS. At present, intracoronary ultrasound and coronary angiography can be used for the detection of coronary atherosclerotic plaques. However, these two methods are not widely applied in the clinic as they are expensive and invasive, and require improvements (3).

Estrogen, a female hormone, not only promotes growth and development, but also has a role in various systems of the human body (4). High-sensitivity C-reactive protein (hs-CRP) is produced by hepatocytes and is involved in inflammation. Clinical studies have demonstrated that hs-CRP levels can be used as predictors of cardiovascular disease (5). Matrix metalloproteinase 9 (MMP-9, also known as gelatinase B), which has numerous substrates, is involved in a wide range of physiological functions, including regulation of protease and cytokine activity (6). In addition, MMP-9 plays a role in elastin degradation, which promotes breakdown of the thin, fibrous caps of plaques (7). The aim of the present study was to explore an economic, convenient and non-invasive method to evaluate the extent of coronary artery lesions and to analyze the correlation between estrogen, hs-CRP and MMP-9 levels and the severity of coronary artery lesions.

## Subjects and methods

### Subjects

From March 2011 to March 2012, 65 patients with ACS diagnosed by coronary angiography, including 30 patients with AMI and 35 patients with UAP, were randomly enrolled from the First Affiliated Hospital of Xinxiang Medical University (Weihui, China). A total of 33 patients with stable angina pectoris (SAP) and 36 healthy individuals were also included. Exclusion criteria included hepatic or renal dysfunction, thyroid disease, cancer, autoimmune disease, chronic bronchitis and asthma. The mean age of the patients with AMI (16 male, 14 female) was 61.46±9.37 years, with a range of 51–73 years. The mean age of the patients with UAP (19 male, 16 female) was 60.92±10.79 years, with a range of 49–74 years. The mean age of the patients with SAP (16 male, 17 female) was 61.13±11.05 years, with a range of 50–72 years. The mean age of the healthy subjects (19 male, 17 female) was 61.71±11.88 years, with a range of 49–74 years. There were no significant differences in age and gender among the four groups (P>0.05). This study was conducted in accordance with the Declaration of Helsinki, and approval was obtained from the Ethics Committee of the First Affiliated Hospital of Xinxiang Medical University. Written informed consent was obtained from all subjects

### Methods

Venous blood samples (4 ml) were obtained from all subjects following an overnight fast. The blood samples were centrifuged at 5,000 × g for 10 min and the supernatants were stored at -30°C. ELISA was used to detect serum levels of estrogen, hs-CRP and MMP-9 (8–10). Coronary angiography was performed in all patients, including healthy subjects, according to the Judkins technique (11). Two experienced interventional cardiologists assessed the extent of coronary artery lesions and the number of stenosed coronary vessels. To assess the extent of coronary artery lesions, the diameter of the proximal coronary artery was measured. Patients were classified as having mild (50–70% lesion), moderate (71–90% lesion), severe (91–99% lesion) or total occlusion (100% lesion). Coronary artery stenosis was defined as ≥50% narrowing of vessels Left main coronary artery stenosis was calculated as two-vessel coronary artery stenosis.

### Statistical analysis

Version 17.0 of the SPSS statistical software package (SPSS, Inc., Chicago, IL, USA) was used for all statistical analysis. Multiple groups were compared using analysis of variance. Correlations were analyzed using linear correlation analysis and Spearman’s rank correlation analysis. P<0.05 was considered to indicate a statistically significant difference.

## Results

### Comparison of serum levels of estrogen, hs-CRP and MMP-9

Serum estrogen levels were significantly lower in patients with AMI (52.31±8.94 ng/l), UAP (58.72±8.21 ng/l) and SAP (61.93±8.69 ng/l) compared with the control group (82.16±9.85 ng/l; P<0.05). Estrogen levels were also significantly different among the AMI, UAP and SAP groups (P<0.05). Compared with the control group (2.31±1.54 mg/l), the AMI (7.16±2.82 mg/l), UAP (5.77±2.33 mg/l) and SAP groups (3.38±1.72 mg/l) had significantly higher serum hs-CRP levels (P<0.05). Serum hs-CRP levels were also significantly different among the AMI, UAP and SAP groups (P<0.05). Serum MMP-9 levels were significantly higher in patients with AMI (347.86±22.58 ng/ml), UAP (283.95±19.51 ng/ml) and SAP (195.32±18.46 ng/ml) compared with the control group (123.89±18.08 ng/ml; P<0.05). MMP-9 levels were also significantly different among the AMI, UAP and SAP groups (P<0.05) (Table I).

### Correlation analysis between serum levels of estrogen, hs-CRP and MMP-9

**Figure 1:**
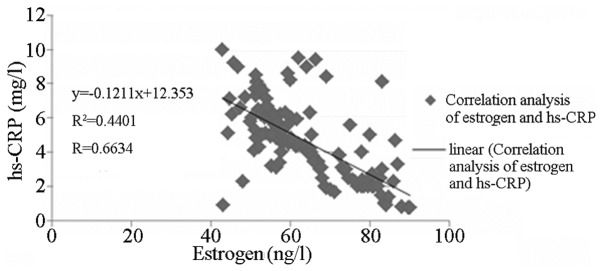
Correlation between serum levels of estrogen and high-sensitivity C-reactive protein (hs-CRP).

**Figure 2:**
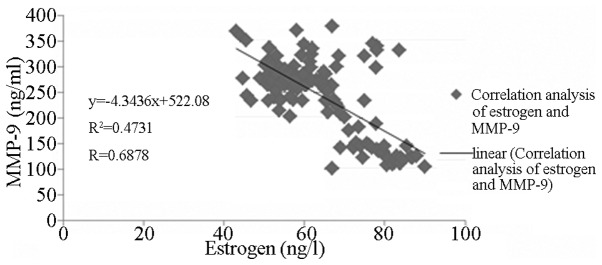
Correlation between serum levels of estrogen and matrix metalloproteinase 9 (MMP-9).

**Figure 3:**
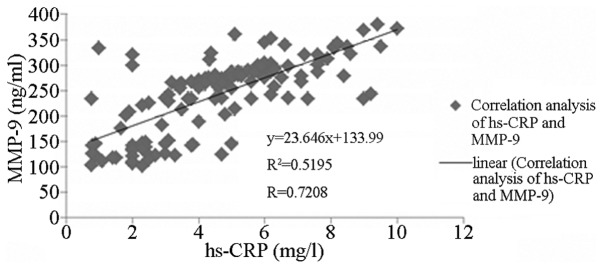
Correlation between serum levels of high-sensitivity C-reactive protein (hs-CRP) and matrix metalloproteinase 9 (MMP-9).

As shown in Figs. 1 and 2, serum estrogen levels were negatively correlated with hs-CRP and MMP-9 levels (r=−0.6634 and −0.6878, respectively; both P<0.05). As shown in Fig. 3, hs-CRP and MMP-9 levels correlated positively (r=0.7208, P<0.05).

### Correlation analysis between the number of stenosed coronary vessels and serum levels of estrogen, hs-CRP and MMP-9

Estrogen levels were significantly different among patients with one-, two- and three-vessel disease. The number of stenosed coronary vessels correlated negatively with estrogen levels (r=-0.6467, P<0.05) and positively with hs-CRP and MMP-9 levels (r=0.6519 and 0.6835, respectively; both P<0.05) (Table II).

## Discussion

ACS describes a spectrum of clinical symptoms that result from acute myocardial ischemia, including AMI and UAP (12). It has been indicated that instability of coronary atherosclerotic plaques plays an important role in the pathogenesis of ACS. Erosion or rupture of coronary plaques, leading to partial or complete obstruction of the coronary artery, results in AMI or angina pectoris (13,14). In clinical practice, early diagnosis of the stability of plaques and the severity of coronary artery lesions is important for patients with ACS. As current diagnostic approaches for ACS are invasive and expensive, non-invasive and cost-efficient methods for the diagnosis of ACS are being widely explored. At present, few studies have investigated the correlation between severity of coronary artery lesions and serum levels of estrogen, hs-CRP and MMP-9. This study investigated the correlation between the severity of coronary artery lesions and serum levels of estrogen, hs-CRP and MMP-9 in patients with ACS.

Estrogen, an important hormone, possesses a wide spectrum of biological activities. In addition to its role in reproduction, estrogen affects the endocrine, cardiovascular and metabolic systems. It has been observed that the incidence of coronary heart disease in pre-menopausal females is significantly lower than that in post-menopausal females and males (15). In the present study, serum estrogen levels in patients with coronary artery disease were significantly lower than those in healthy subjects. Serum estrogen levels progressively increased across the AMI, UAP and SAP groups, respectively, while a progressive decrease occurred across the one-, two- and three-vessel disease subgroups, respectively. hs-CRP, an indicator of inflammatory states, is involved in the pathogenesis of atherosclerosis. Levels of hs-CRP change with coronary artery plaque characteristics, and hs-CRP is also a predictor of coronary artery disease-associated mortality (16). MMP-9, an important member of the matrix metalloproteinase family, is involved in the invasion and metastasis of cancer cells, inflammation and angiogenesis, as well as the initiation and development of atherosclerosis (17). Park *et al* (18) demonstrated that MMP-9 levels were significantly higher in patients with vulnerable atherosclerotic plaques than in patients without vulnerable atherosclerotic plaques, indicating that MMP-9 levels were closely associated with vulnerable atherosclerotic plaques. In the present study, highest levels of hs-CRP and MMP-9 were observed in patients with AMI, followed by those with UAP and SAP, respectively. Levels of hs-CRP and MMP-9 progressively increased across the one-, two- and three-vessel disease groups. Correlation analyses demonstrated that levels of estrogen, hs-CRP and MMP-9 were significantly correlated with the number of stenosed coronary vessels, and there was also a correlation between levels of estrogen, hs-CRP and MMP-9. These results indicate that levels of estrogen, hs-CRP and MMP-9 are associated with the severity of coronary artery lesions and the stability of coronary artery plaques in patients with ACS, which is consistent with previous studies (17–19). Compared with patients with SAP, patients with ACS had a greater number of vulnerable and unstable atherosclerotic plaques in which inflammation was more active.

In conclusion, there is a significant correlation between levels of estrogen, hs-CRP and MMP-9, which can be used as biomarkers to evaluate the severity of coronary artery lesions and to predict the stability of coronary artery plaques.

## Figures and Tables

**Table I:** Serum levels of estrogen, hs-CRP and MMP-9 in the AMI, UAP, SAP and control groups.

Groups	Estrogen (ng/l)	hs-CRP (mg/l)	MMP-9 (ng/ml)
AMI (n=30)	52.31±8.94^a^–^c^	7.16±2.82^a^–^c^	347.86±22.58^a^–^c^
UAP (n=35)	58.72±8.21^a^,^b^	5.77±2.33^a^,^b^	283.95±19.51^a^,^b^
SAP (n=33)	61.93±8.69^a^	3.38±1.72^a^	195.32±18.46^a^
Control (n=36)	82.16±9.85	2.31±1.54	123.89±18.08

Results are presented as the mean ± standard deviation.

a P<0.05 compared with the control group;

b P<0.05 compared with the SAP group;

c P<0.05 compared with the UAP group.

AMI, acute myocardial infarction; UAP, unstable angina pectoris; SAP, stable angina pectoris; hs-CRP, high-sensitivity C-reactive protein; MMP-9, matrix metalloproteinase 9.

**Table II:** Serum levels of estrogen, hs-CRP and MMP-9 in patients with one-, two- and three-vessel disease.

Disease type	Estrogen (ng/l)	hs-CRP (mg/l)	MMP-9 (ng/ml)
One-vessel (n=12)	56.04±8.80	3.63±1.54	236.24±19.50
Two-vessel (n=29)	53.72±9.56^a^	5.84±1.79^a^	268.54±19.31^a^
Three-vessel (n=24)	49.87±9.06^a^,^b^	7.47±2.36^a^,^b^	302.73±20.62^a^,^b^

Results are presented as the mean ± standard deviation.

a P<0.05 compared with the one-vessel disease group;

b P<0.05 compared with the two-vessel disease group.

hs-CRP, high-sensitivity C-reactive protein; MMP-9, matrix metalloproteinase 9.
